# Natural Nanoparticles: A Safe Bullet for Treatment and Detection of Solid Tumors

**DOI:** 10.3390/pharmaceutics14061126

**Published:** 2022-05-25

**Authors:** Serena Mazzucchelli

**Affiliations:** Dipartimento di Scienze Biomediche e Cliniche, Università di Milano, 20157 Milan, Italy; serena.mazzucchelli@unimi.it

In the last couple of decades, nanoparticles have been extensively studied as carriers for cancer imaging agents and as drug delivery platforms, due to their ability to positively affect the distribution and tumor-targeting properties of delivered compounds [1,2]. However, nanoparticle off-target accumulation, immunogenicity, and sequestration by macrophages are issues that strongly limit their clinical translation [3,4]. These issues could be overcome by the use of natural nanoparticles, since they are mainly constituted by proteins or by cell membrane portions, resulting in high biocompatibility, stability in biological fluids and bioinvisibility [5]. This Special Issue depicts the current landscape of natural nanoparticles for cancer diagnosis and treatment, collecting articles able to better highlight any feature relating to this topic. Not only natural nanoparticles, such as exosomes and protein nanocages, but also organic nanoformulations of natural compounds, such as the flavonoid luteolin and the xanthone derivate α-mangostin [6,7,8,9], represent attractive options to improve knowledge in this area and to accelerate the translation of nanotechnological products to the clinic, as summarized by Ion. D. et al. [10]. Among natural nanoparticles, ferritins (HFn) are the most fascinating natural drug delivery system, and have been extensively investigated in relation to cancer due to their intrinsic tumor-targeting capability, as reported by Mainini F. et al., in this Special Issue [11]. HFn display the targeted recognition of cancer cells and tissues thanks to their ability to specifically recognize the transferrin receptor 1 (TfR1), which is overexpressed in tumors [11]. HFn-based nanoparticles have been engineered by DNA recombinant technology or by chemical surface modification, obtaining products exploitable for the treatment, diagnosis and theranostics of cancer, already tested both in vitro and in vivo with very promising results [11]. In addition to ferritin-based nanoparticles, an important position is occupied by exosomes, a class of natural nanosized vesicles derived by cells for intracellular communication and recently developed and assessed for drug delivery purposes [12]. They display a typical tropism determined by the cell subtype from which they are generated. Here, we report a study about exosomes from M1 macrophages loaded with the combination of gemcitabine and the oral iron chelator deferasirox, developed and tested for the treatment of gemcitabine-resistant pancreatic cancer [13]. This work demonstrated how drug entrapment into M1-macrophage-derived exosomes is an effective strategy to bypass drug resistance in this pancreatic cancer model, suggesting that it could be successfully exploited for resistant pancreatic cancer and investigated in other cancer subtypes [13]. To date, the clinical exploitation of exosomes derived from human or mammalian sources is challenging, since the set-up of large-scale human or mammalian cultures to produce exosomes seems to limit the feasibility [14]. Recently, exosomes derived from the budding of bacterial membranes have been explored as a valuable alternative for large-scale productions [14]. The review of S. Fazal and R. Lee describes bacterial membrane vesicles (BMV), focusing attention on the sources used for their production and on techniques for separation, purification, characterization and drug loading. Moreover, also discussed was their exploitation as drug delivery platforms in cancer therapy, with some in vivo applications reported [14]. Finally, this Special Issue is completed by a literature review of nanotechnological solutions adopted for the delivery of RNA-i therapeutics in pancreatic cancers [15] and by natural carbon nanodots [16], a field of study that creates a bridge between synthetic and natural nanoparticles, since it employs classical chemical methods of synthesis starting from natural carbon sources [16].

Overall, the articles and reviews collected in this Special Issue describe a prolific and dynamic field of study, which promises to bring natural nanocarriers to cancer clinical trials, accelerating clinical translation.
